# Plasma channel undulator excited by high-order laser modes

**DOI:** 10.1038/s41598-017-16971-5

**Published:** 2017-12-04

**Authors:** J. W. Wang, C. B. Schroeder, R. Li, M. Zepf, S. G. Rykovanov

**Affiliations:** 1 Helmholtz Institute Jena, Fröbelstieg 3, Jena, 07743 Germany; 2 Lawrence Berkeley National Laboratory, 1 Cyclotron Road, Berkeley, California 94720 USA; 3 Shanghai Institute of Optics and Fine Mechanics, Chinese Academy of Sciences, Shanghai, China; 4 Institut für Optik und Quantenelektronik, Friedrich-Schiller-Universität Jena, Max-Wien-Platz 1, Jena, 07743 Germany; 5 Department of Physics and Astronomy, Queen’s University Belfast, Belfast, BT7 1NN UK

## Abstract

The possibility of utilizing plasma undulators and plasma accelerators to produce compact ultraviolet and X-ray sources, has attracted considerable interest for a few decades. This interest has been driven by the great potential to decrease the threshold for accessing such sources, which are mainly provided by a few dedicated large-scale synchrotron or free-electron laser (FEL) facilities. However, the broad radiation bandwidth of such plasma devices limits the source brightness and makes it difficult for the FEL instability to develop. Here, using multi-dimensional particle-in-cell (PIC) simulations, we demonstrate that a plasma undulator generated by the beating of a mixture of high-order laser modes propagating inside a plasma channel, leads to a few percent radiation bandwidth. The strength of the undulator can reach unity, the period can be less than a millimeter, and the number of undulator periods can be significantly increased by a phase locking technique based on the longitudinal tapering. Polarization control of such an undulator can be achieved by appropriately choosing the phase of the modes. According to our results, in the fully beam loaded regime, the electron current in the plasma undulator can reach 0.3 kA level, making such an undulator a potential candidate towards a table-top FEL.

## Introduction

X-ray radiation sources have important applications in biology, medicine, industry and fundamental science because of their capability of resolving the structure and dynamics of matter on the molecular and atomic scales^1–3^. For the past twenty years, the third-generation synchrotron facilities have been the workhorse X-ray sources^4^. By the virtue of an electron beam instability called microbunching^5^, much brighter, shorter, and fully coherent XUV and X-ray pulses can be generated in the devices called free-electron lasers (FELs)^5–7^. The excellent FEL radiation properties make it possible to time-resolve molecular structural dynamics and obtain high-resolution images. However, synchrotron and FEL facilities are typically large, expensive, and oversubscribed for the users.

It has been demonstrated that schemes based on laser-plasma interaction are able to produce ultrashort and bright radiation^8–16^. In a typical scenario, electrons accelerated by a laser-excited wakefield^17,18^, oscillate either in the focusing fields of the wake or inside an (external) undulator, emitting bright radiation with a fundamental wavelength of $\lambda ={\lambda }_{u}\mathrm{(1}+{K}^{2}\mathrm{/2)/(2}{\gamma }_{0}^{2})$ on axis, where *λ*_*u*_ is the undulator wavelength, *γ*_0_ is the Lorentz factor of the electron, and *K* is the so-called undulator strength parameter. Compared to a conventional magnetic undulator^12,13^, the period of a plasma-based undulator is short and can be less than a millimeter. Therefore, a laser-created plasma undulator together with a laser-plasma electron accelerator (LPA) make it possible to construct an economical and compact incoherent XUV or X-ray source for university laboratories, hospitals, and even commercial applications^15,19^. Plasma undulators can be realized by ultra-intense laser plasma interactions in the bubble regime^8–10^, a laser pulse propagating in plasma perpendicularly to the electron beam propagation direction^20^, a laser pulse interaction with a nanowire array^21^, or using laser pulse offset injection in a matched plasma channel^22–24^. However, it is still an open question whether these plasma undulators can be used as an FEL. In general, not all undulators are suitable for the onset of the FEL instability. For example, in magnetic undulators matching and field uniformity are critical. In the case of plasma undulators one of the major challenges is the large radiation spread caused by varying values of undulator strength *K* throughout the beam or by strong focusing and hence large electron beam divergence inside the wakefield, while for the FEL process to develop a very narrow bandwidth is required^8,15^. Another challenge is that the phase slippage between the electrons and the wakefield limits the length of a plasma undulator. Furthermore, it is not generally the case in plasma undulators that the electron trajectories are independent of the injection positions. We show for the first time that a solution exists for a plasma undulator that meets the stringent conditions required for the FEL lasing.

Here we propose a phase-locked plasma undulator created by the wakefields of a combination of high-order laser modes propagating in a parabolic plasma channel. We demonstrate that the undulator fields can be made uniform along the transverse direction by choosing appropriate intensities of the laser modes. This enables all the electrons to oscillate with the same strength parameter *K* on the order of unity for a few tens of undulator periods. The plasma density in the channel can be tapered to lock the phase between the electrons and the wakefield, which significantly increases the number of electron oscillations, i.e. the total undulator length. As a result, X-ray radiation with high brightness and narrow bandwidth is generated when a high-energy electron beam is injected into the present plasma undulator. The beam loading limit indicates that the current of the beam can reach approximately 0.3 kilo-Ampere for typical laser-plasma parameters. These properties imply that such a plasma undulator is a miniaturised electron device naturally matching the extremely compact scale of a plasma accelerator, similar in significance to the recent breakthrough development of plasma lenses^25–27^, and plasma accelerator staging^28,29^, and may have great potential in incoherent XUV and X-ray sources or future compact FELs.

## Results

### Principles of the plasma channel undulator

**Figure 1:**
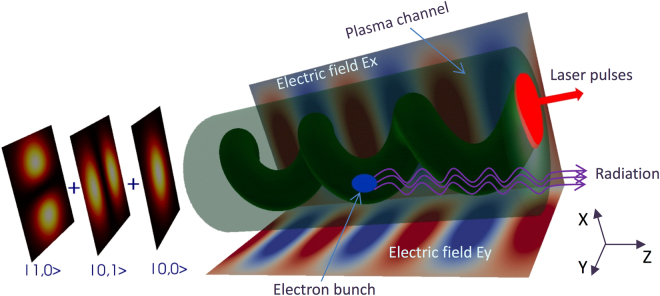
Schematic of the plasma channel undulator. As an example, three *y*-polarized Hermite-Gaussian modes, |0, 0〉, |0, 1〉, and |1, 0〉, propagate in a matched parabolic plasma channel. In both the (*x*, *z*) plane and the (*y*, *z*) plane, the profile of the total intensity oscillates with a wavelength proportional to the Rayleigh length of the pulse, and can be controlled by choosing the mode numbers. The ellipticity of the laser pulse oscillations can be controlled by the phase difference between the modes |0, 1〉 and |1, 0〉. The focusing fields of the induced plasma wakefield serve as an undulator. Electrons injected into the wakefield will experience undulator oscillations and emit bright radiation.

Laser pulse guiding for distances much larger than the Rayleigh length of the laser pulse are necessary in LPAs for achieving GeV level of electron energy. Typically a plasma channel that has a parabolic (or close to parabolic) transverse density distribution is used in LPA experiments for laser guiding^30^. For laser power below the critical power^18^, laser pulses with transverse profile given by Hermite-Gaussian (or Laguerre-Gaussian) modes will propagate inside the channel without changing their transverse shape given that their spotsize is matched to the channel radius. Plasma undulator can be generated by propagating a mixture of different Hermite-Gaussian laser modes in a matched plasma channel, as shown by the schematic in Fig. 1. An oscillatory behaviour of intensity envelope appears when the modes with the same polarization co-propagate in the plasma channel, since the phase velocities for different modes are dependent on the mode numbers. For example, the wavelength of the oscillations of the total intensity of a mixture of two modes |*m*_0_, *p*_0_〉 and |*m*_1_, *p*_1_〉 is 2*πZ*_*R*_/|(*m*_0_ + *p*_0_) − (*m*_1_ + *p*_1_)|^31^, where the quantum ket notation |*m*, *p*〉 indicates a Hermite-Gaussian mode with an order number *m* in *x* direction and an order number *p* in *y* direction, and ${Z}_{R}=\pi {w}_{0}^{2}/{\lambda }_{L}$ is the Rayleigh length with *w*_0_ the laser spot size and *λ*_*L*_ the wavelength of the laser pulses, which is assumed to be the same for all the modes. It is also important to mention that to obtain the desired undulator fields one has to create an asymmetric initial transverse intensity distribution of the modes mixture, which is achieved by mixing even and odd mode numbers. The wakefield generated by the two modes will also oscillate while propagating in the channel, which provides an additional control of the focusing field. Consider a two-dimensional case, then the transverse wakefield generated by two *y*-polarized modes *m*_0_ and *m*_1_ can be expressed as (see Methods for the calculation of the wakefield)

1 $${E}_{x}=\frac{{E}_{0}C}{{k}_{p}}[\frac{4{\alpha }_{\beta }x}{{w}_{0}^{2}}+\frac{4{\alpha }_{u}}{{w}_{0}}\,\cos \,\frac{z({m}_{0}-{m}_{1})}{{Z}_{R}}]\,\sin ({k}_{p}\zeta ),$$

with

2 $${\alpha }_{\beta }=\frac{-{a}_{{m}_{0}}^{2}(2{m}_{0}+1)}{{m}_{0}{!2}^{{m}_{0}}}{[\frac{{m}_{0}!}{(\frac{{m}_{0}}{2})!}]}^{2}+\frac{4{a}_{{m}_{1}}^{2}}{{m}_{1}{!2}^{{m}_{1}}}{[\frac{{m}_{1}!}{(\frac{{m}_{1}-1}{2})!}]}^{2},$$

and

3 $${\alpha }_{u}=\frac{{a}_{{m}_{0}}{a}_{{m}_{1}}\sqrt{{m}_{0}!{m}_{1}!}}{\sqrt{{2}^{{m}_{0}}{2}^{{m}_{1}-1}}}\frac{{(-\mathrm{1)}}^{({m}_{0}+{m}_{1}-\mathrm{1)/2}}}{\frac{{m}_{0}}{2}!\frac{{m}_{1}-1}{2}!},$$

where *ζ* = *z* − *ct*, $C=(\sqrt{\pi }{k}_{p}L\mathrm{/4)}\exp (-{k}_{p}^{2}{L}^{2}\mathrm{/4)}$, *E*_0_ = *m*_*e*_ *c* ^2^ *k*_*p*_/*e*, *L* is the laser pulse longitudinal length, *k*_*p*_ is the plasma wave number, *e* is the electron charge, *m*_*e*_ is the electron mass and *c* is the light speed in vacuum. As can be seen from equation (1), the transverse wakefield is separated into two parts: the betatron part which is linearly proportional to the transverse coordinate *x*, and the undulator part which is a simple cosine function of the longitudinal coordinate *z*. The strengths of these two parts *α*_*β*_ and *α*_*u*_ are determined by both the laser modes intensities and order numbers. Electrons with Lorentz factor *γ*_0_ injected in such a wakefield will experience two kinds of oscillations: betatron oscillations with a wave number ${k}_{\beta }=\sqrt{\mathrm{4|}{a}_{\beta }|C/{\gamma }_{0}{w}_{0}^{2}}$ and undulator oscillations with a wave number *k*_*u*_ = |*m*_0_ − *m*_1_|/*Z*_*R*_. Such a plasma wakefield structure serves as an undulator (or wiggler) and leads to electron oscillations and generation of synchrotron radiation. In contrast to the plasma undulator generated by an off-axis injected laser pulse^22^, in which case it is difficult to suppress the strong focusing field (large *α*_*β*_), in the plasma undulator created by high-order modes one can eliminate the betatron part by choosing appropriate laser intensities *a*_*m*_ and *a*_*n*_ to satisfy *α*_*β*_ = 0 in equation (2). Physically, the focusing field disappears because the sum of the intensities of the two modes keeps constant near the axis, and thus the transverse gradient of the potential reduces to zero. As a result, electrons injected into such a particular wakefield will oscillate only with the undulator frequency *k*_*u*_. This significantly enhances the undulator radiation, which is more interesting because its energy can be located in the soft or even hard X-ray range and its radiation bandwidth is very narrow. It is also worth noting that the undulator strength parameter here *K* = 4*πCα*_*u*_ *w*_0_/*λ*_*L*_, is independent of the electron transverse positions, unlike the typical betatron case^8–10,14^. This property is helpful for decreasing the undulator radiation bandwidth, which should be smaller than the Pierce parameter *ρ* for the FEL application^5,8,15^.

### Electron dynamics and radiation

**Figure 2:**
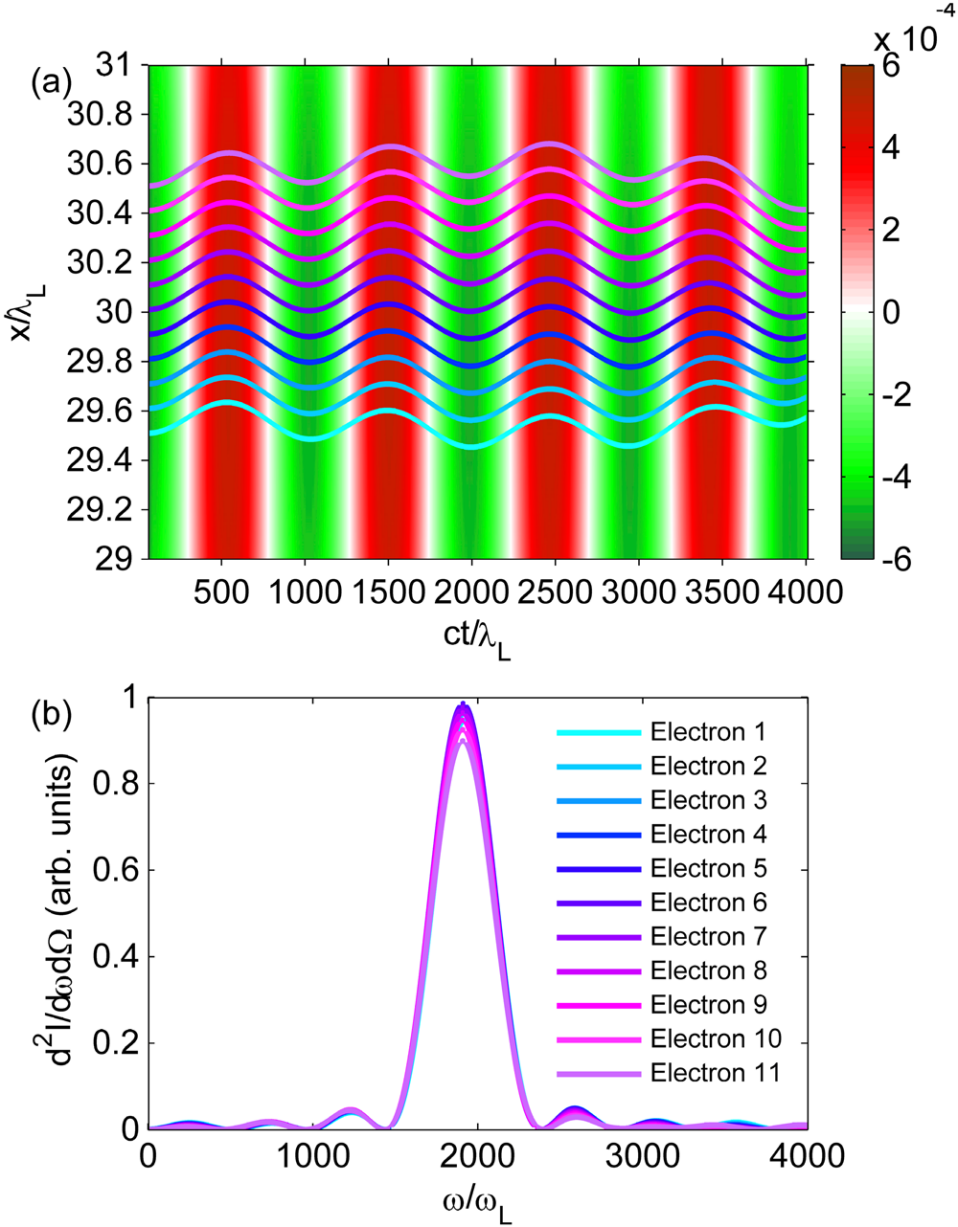
Distribution of the undulator field, test electron trajectories and their on-axis radiation spectrum. (**a**) The distribution of the transverse electric field normalized by *mc* ^2^ *k*_*p*_/*e* in the space (*x*, *ct*) for a fixed position *k*_*p*_ *ζ* = −5*π*/2. The wakefield is created by two linearly polarized modes *m*_0_ = 0 and *m*_1_ = 1, with intensities *a*_0_ = 0.14 and *a*_1_ = 0.1, spot radius *w*_0_ = 7*λ*_*L*_, and duration *τ* = 15*T*_*L*_, in a plasma channel with an on-axis density of 0.001*n*_*c*_, where ${n}_{c}={m}_{e}{\omega }_{L}^{2}\mathrm{/(4}\pi {e}^{2})$ is the critical plasma density with *ω*_*L*_ the laser frequency. The blue lines represent the trajectories of the test electrons. (**b**) The corresponding on-axis radiation spectra for the test electrons with different initial transverse positions. Only the first harmonic is considered here.

The transverse electric field experienced by the electrons and the trajectories of test electrons from 2D PIC simulations are presented in Fig. 2(a). In the simulation, the fundamental Gaussian mode (*a*_0_ = 0.14) and the first-order Hermite-Gaussian mode (*a*_1_ = 0.1) co-propagate in a matched plasma channel. Detailed parameters are given in the Methods. One can see that the transverse field is periodic with time and almost uniform in transverse coordinate *x* in a range of 2*λ*_*L*_, analogous to the distribution of the magnetic field in a magnetic undulator^32^. The trajectories of the test electrons are almost the same, although they are injected into the undulator with different initial transverse positions, which is completely different from the case of electrons undergoing betatron oscillations in a plasma wakefield^8–10^. As a result, the on-axis radiation spectra of the test electrons, shown in Fig. 2(b) are almost identical. The frequency of the first harmonic of the on-axis radiation is located at $\omega =2{\gamma }_{0}^{2}{\omega }_{u}\mathrm{/(1}+{K}^{2}\mathrm{/2)}\sim 1900\,{\omega }_{L}$, where *K *= 0.44 is the strength parameter and *ω*_*u*_ = *c*/*Z*_*R*_ is the undulator frequency, corresponding to approximately millimeter undulator period. For *λ*_*L*_ = 1 *μ*m, the radiation wavelength is 0.5 n*m*, which is in the soft x-ray range. Theoretically, higher photon energies, even reaching the hard X-ray region, can be achieved by co-propagating even higher order modes together with the fundamental Gaussian mode. The undulator strength parameter *K* can be increased by changing the intensities of the laser pulses. In the above discussions the injected electrons are assumed to be locked at a certain phase. However, such condition can not be satisfied in most cases, because the injected high-energy electrons always run faster than the wakefield, which is referred to as dephasing in laser wakefield acceleration^18^. For an electron initially injected into the phase *k*_*p*_ *ζ* = −5*π*/2, its phase will slip forward to *k*_*p*_ *ζ* = −2*π* after a dephasing length *L*_*D*_. For a highly relativistic electron ${\upsilon }_{z}\simeq c$ traveling in a plasma channel, the dephasing length is given by *L*_*D*_ = *λ*_*p*_/[4(1 − *υ*_*p*_/*c*)], where ${\upsilon }_{p}=c\sqrt{1-{\omega }_{p}^{2}/{\omega }_{L}^{2}-\mathrm{(4}{c}^{2})/({\omega }_{L}^{2}{w}_{0}^{2})}$ is approximately the phase velocity of the plasma wave, *ω*_*L*_ is the laser frequency, *ω*_*p*_ is the plasma frequency and *λ*_*p*_ is the plasma wavelength. In the simulation presented in Fig. 2, the on-axis plasma density is *n*_*p*0_ = 0.001 *n*_*c*_ and electron initial energy *γ*_0_ = 1000, leading to the dephasing length *L*_*D*_ ≈ 5000*λ*_*L*_. Dephasing sets a limitation to the number of undulator periods and, hence, reduces the radiation brightness. Moreover, varying of the electron energy broadens the radiation spectrum. This could be a limitation should this undulator be used for a compact FEL. In order to avoid dephasing and obtain radiation with high brightness and narrow-band spectrum, it is necessary to lock the phase of the electrons in the wakefield. In this work, phase-locking by longitudinally tapering the plasma channel is used (see Methods)^33^. As the electrons slip forward with respect to the driver laser pulses, the plasma density is increased, reducing the plasma wavelength and maintaining the phase of the electrons inside the plasma wave bucket.

**Figure 3:**
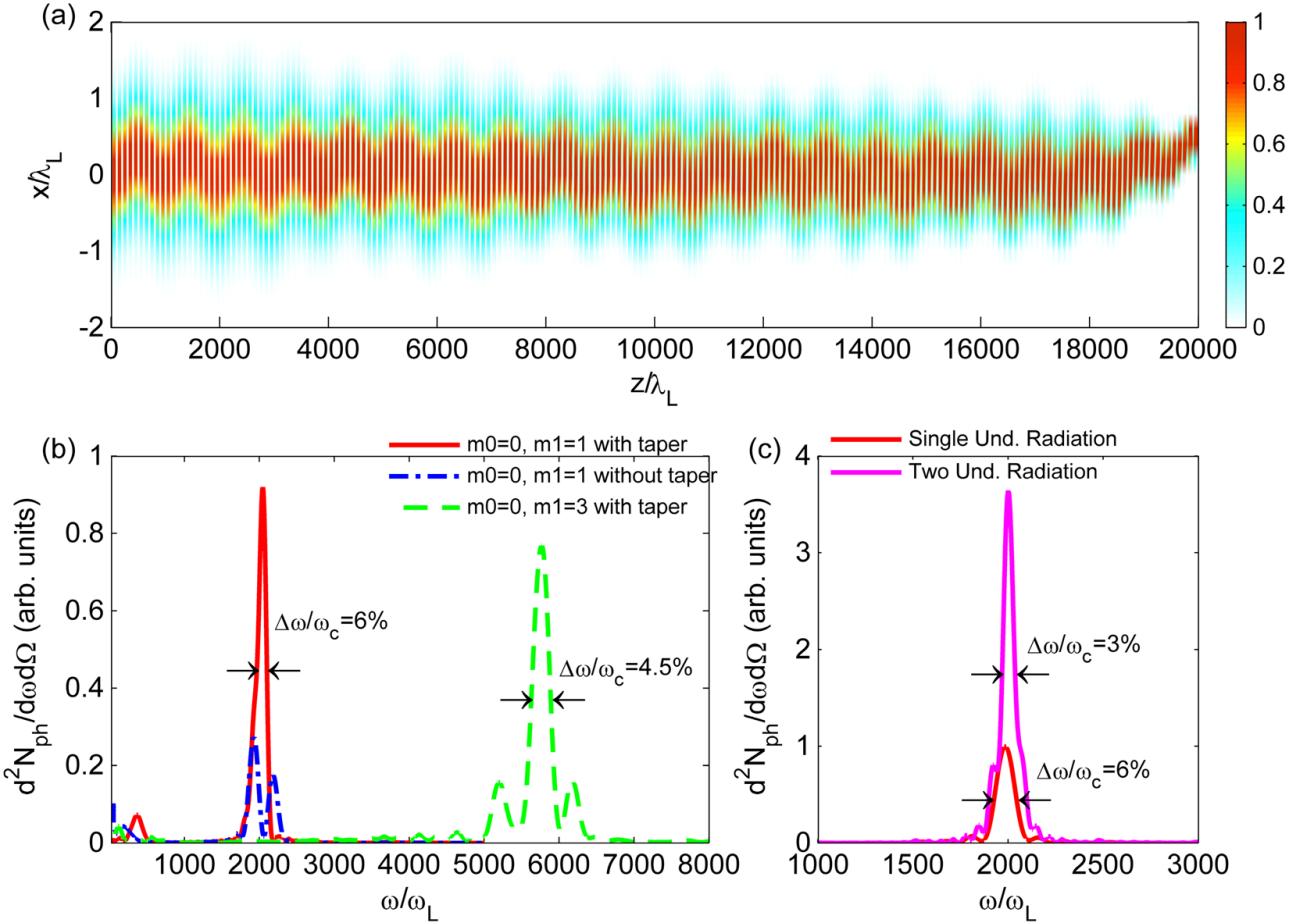
Trajectory and radiation spectrum of the electron beam propagating through the plasma undulator(s). (**a**) The trajectory of the beam in the plasma undulator. The electron density of the beam has been normalized. The parameters of the laser pulses and plasma are the same as in Fig. 2 except that the plasma density has been tapered to lock the phase of the electrons inside the plasma wakefield. (**b**) The on-axis radiation spectra of the electron beam with taper (red solid line) and without taper (blue dash-dot line) using modes *m*_0_ = 0 and *m*_1_ = 1. The green dashed line corresponds to the case of the modes *m*_0_ = 0 and *m*_1_ = 3 with taper. (**c**) The on-axis radiation spectra from a single undulator (red line) and two phase-matched undulators (magenta line).

We now investigate the dynamics of an electron beam propagating in the phase-locked plasma undulator. Parameters of the electron beam can be found in the Methods. The evolution of the beam is shown in Fig. 3(a). One can see that the beam keeps a constant radius while oscillating in the undulator with the wavelength *λ*_*u*_. Because the plasma channel has been tapered to lock the phase of the beam in the wakefield, the electron beam can stably propagate in the undulator for approximately 20000 *λ*_*L*_, corresponding to the number of undulator periods *N*_*u*_ = 20. The radiation spectrum emitted by the beam is shown with the red solid line in Fig. 3(b). The spectrum bandwidth is as narrow as 6%, which is very close to the theoretical bandwidth $\sqrt{{(1/{N}_{u})}^{2}+{(2{\rm{\Delta }}\gamma /{\gamma }_{0})}^{2}+{({\gamma }_{0}{\rm{\Delta }}{\theta })}^{4}/16}$ ^34^, where ${\rm{\Delta }}\gamma \simeq 2.35\,{\sigma }_{\gamma }$ is the FWHM electron beam energy spread, and $\Delta \theta \simeq 2.35\,{\sigma }_{\theta }$ is the FWHM electron beam angular spread. Also minor betatron radiation located at *ω* = 400*ω*_*L*_ can be seen in the spectrum, which comes from the electrons far from the axis where *α*_*β*_ ≠ 0. The radiation of an electron beam in an untapered plasma channel is also presented with the blue dash-dot line in Fig. 3(b). The spectrum exhibits a two-peak structure with a larger bandwidth and a much lower brightness. This is because the un-phase-locked electrons oscillate in two different plasma wave buckets, then escape from the undulator with an angle and become lost finally. As we stated before, higher frequency radiation can be obtained by using higher order mode mixed with the fundamental mode. The green dashed line in Fig. 3(b) shows the radiation spectrum of the same electron beam in an undulator generated by modes *m*_0_ = 0 and *m*_1_ = 3. One can see that the central frequency of the radiation has increased to 5750 *ω*_*L*_, which is approaching the hard X-ray region. The bandwidth becomes even narrower because the electron beam experiences more undulator periods during the same propagation length.

**Figure 4:**
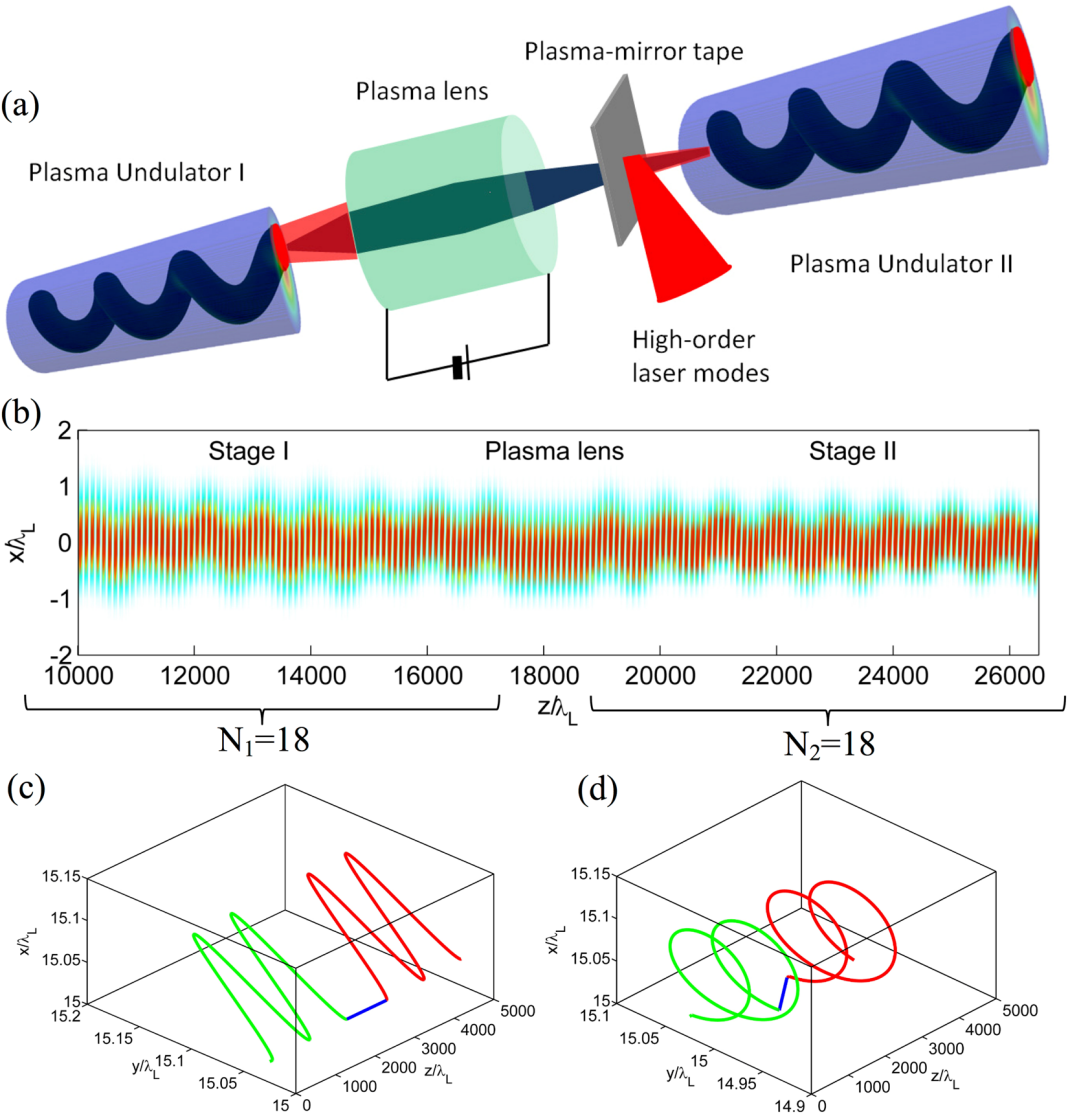
Staging of two plasma undulators using a plasma lens. (**a**) Scheme of two-stage undulator system. The electron beam from the first undulator is transported to the second undulator by a plasma lens. The second plasma undulator is created by the second pair of high-order laser modes which are injected via a plasma-mirror tape. The distance of the two undulators is carefully designed to preserve the optical phase of the electrons. (**b**) The trajectory of an electron beam in the two-stage plasma undulator. Each undulator has 18 periods. (**c**) A typical trajectory of an electron in a 3D two-stage linear undulator. (**d**) A typical trajectory of an electron in a 3D two-stage circular undulator.

It should be mentioned that tapering does not remove the mode slippage that appears due to different group velocities of the laser modes, and can become a main factor limiting the total undulator length. One can estimate the mode slippage length as the length it takes for two modes with numbers *m*_0_ and *m*_1_ to be separated by the longitudinal length *L* of each of the modes; hence, the mode slippage length is ${L}_{m,d}\simeq L{({k}_{L}{w}_{0})}^{2}/(2|{m}_{1}-{m}_{0}|)$. For the parameters of our simulations, this estimate gives the mode slippage length roughly equal to 14 mm, close to the 20 mm slippage length obtained from the numerical simulations. One solution for increasing the mode slippage length and extending the overlap of the laser modes is to employ longer pulses albeit with higher intensities. For the applications in FELs, the required number of periods is on the order of 1/*ρ* ($\rho \sim 0.001$), which is still much larger than that of the plasma undulator. A multiple-stage scheme could be considered to extend the number of periods. In Fig. 4(a) a schematic for a staged plasma undulator system is presented. The electron beam from the first undulator is transported to the second undulator by a discharge capillary, which acts as an active plasma lens^25,28^. An azimuthal focusing magnetic field is produced when an axial discharge current is introduced in the gas-filled capillary. It was reported that the field gradient could be larger than 3000 T/m^25^, enabling cm-scale focal length for GeV-level beam energies. The second undulator can be excited by the second pair of high-order laser modes reflected by a tape-based plasma mirror^28^. The trajectory of the electron beam in the two-stage plasma undulator obtained from PIC simulations is presented in Fig. 4(b). Each stage has 18 periods. Such a two-stage undulator also works in a 3D geometry, as shown by the typical trajectories of one electron in a linear undulator (Fig. 4(c)) and in a circular undulator (Fig. 4(d)). An important point that should be mentioned here is that in order to preserve the phase of the electrons with respect to the radiation field, one needs to carefully design the distance between the two undulators. For an electron with a gamma factor of *γ*_0_, the phase shift after a length *L* in the radiation field is $\delta {\phi }=L\mathrm{/(2}{\gamma }_{0}^{2})$. For proper phase matching *δφ* should be an integral multiple of the radiation wavelength *λ*. For our case here, *γ*_0_ = 1000 and *λ* = 0.53 *nm*, the required distance is *L* = 1.06*η* mm, where *η* is an integer. In the simulation we choose *L* = 1.06 mm and get the on-axis radiation for an electron beam, as shown in Fig. 3(c). The radiation brightness in a two-stage phase-matched undulator is almost 4 times higher than in a single undulator. Also the bandwidth decreases from 6% to 3%. Such a multi-stage scheme provides the possibility to realize an FEL by employing many segments of plasma undulator.

### Radiation polarization control

**Figure 5:**
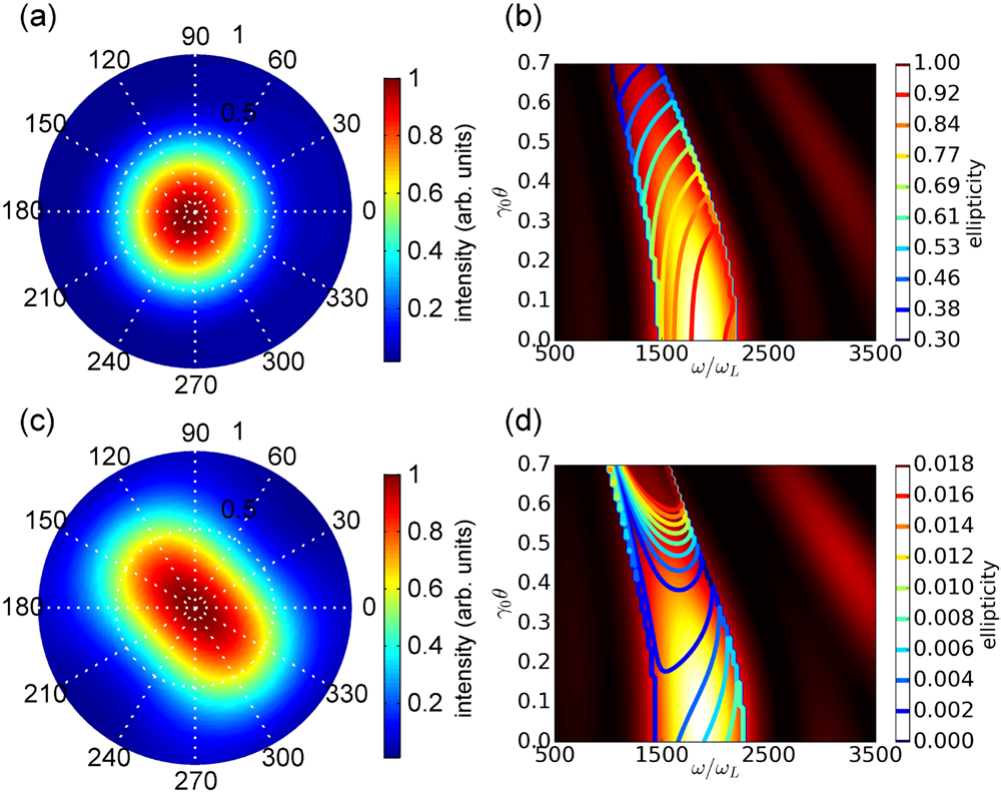
Distributions of the radiation intensity and polarization. The spatial distribution (**a**) and polarization distribution (**b**) of circularly polarized radiation generated when the phase difference between the modes |0,1〉 and |1,0〉 is *π*/2. (**c**) and (**d**) are the spatial distribution and polarization distribution, respectively, for the linearly polarized radiation, generated when the phase difference between the two modes is 0. In figures (**a**) and (**c**), the unit of the polar angle *θ* is milliradian, while the unit of the azimuthal angle *ϕ* is degree. In figures (**b**) and (**d**) the color-coded image represents the normalized energy-angular spectrum, while the contour lines represent the radiation ellipticity, with ellipticity equal to 1 corresponding to circular polarization and 0 corresponding to linear polarization.

The polarization control of the X-ray is motivated by the applications in studying the dynamics of magnetization^35^ and polarization-dependent X-ray absorption spectroscopy^36^. For the present plasma undulator, the flexibility of the radiation polarization can be achieved by controlling the laser pulse phases. Consider three Hermite-Gaussian modes in a three dimensional geometry, |0, 0〉, |0, 1〉 and |1, 0〉, propagating in a matched plasma channel. The mixture of modes |0, 0〉 and |1, 0〉 makes the laser pulse oscillate in the *x* direction, while |0, 0〉 and |0, 1〉 leads to oscillations in the *y* direction. Different phases of these two orthogonal oscillations, determined by the phase difference of the two modes |0, 1〉 and |1, 0〉, and assuming that the amplitudes of these two modes are the same, control the polarization of the laser pulse centroid oscillation, and, hence, the polarization of the wakefield, electron oscillations, and radiation. Figure 5 shows the 3D PIC simulation results of the spatial distribution and polarization distribution of the radiation for the circular and linear polarization cases. Schematically the circular polarization case is presented in Fig. 1. When *φ*_01_ − *φ*_10_ = ±*π*/2, the far field of the radiation concentrates in a circular region with a radius less than one milliradian. Here *φ*_01_ and *φ*_10_ indicate the phase of the mode |0, 1〉 and |1, 0〉, respectively. According to Fig. 5(b), on-axis the radiation is almost perfectly circularly polarized, while it becomes elliptically polarized when the angle *θ* between the observer and the *z* axis increases. Finally, at *θ* = 1/*γ*_0_, the radiation is linearly polarized.

When *φ*_01_ − *φ*_10_ = 0 or *π*, the radiation is linearly polarized, as shown in Fig. 5(d). The spatial distribution also demonstrates a linear structure in Fig. 5(c). In general, fine tunability of the distribution of the radiation intensity and polarization can be obtained by controlling the phases between the modes.

### Beam loading limit and radiation brightness

**Figure 6:**
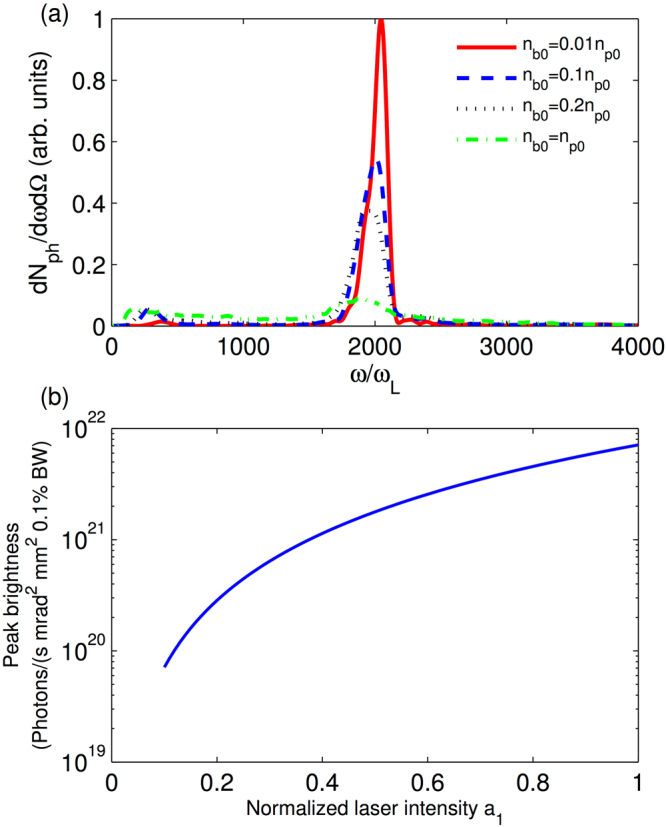
Beam loading effect and brightness scaling. (**a**) The on-axis radiation spectra for different bunch densities: *n*_*b*0_ = 0.01 *n*_*p*0_ (red solid line), *n*_*b*0_ = 0.1 *n*_*p*0_ (blue dashed line), *n*_*b*0_ = 0.2 *n*_*p*0_ (black dotted line), *n*_*b*0_ = 1 *n*_*p*0_ (green dash-dot line). (**b**) The dependence of radiation peak brightness on laser intensity. In the calculation the electron bunch density has been chosen as 1% of the beam loading limit *n*_*bl*_.

A high current electron beam is beneficial for the FEL process^15,32^. For the plasma undulator, one needs to consider the beam loading limit since the electron beam will generate its own wakefield when propagating in the plasma^37,38^. This undesirable wakefield will affect the wakefield generated by the laser pulses, and can even destroy the undulator if it is strong enough. The beam-driven wakefield is zero at the head of the bunch and increases toward the tail. To avoid the breakdown of the undulator, the amplitude of the wakefield generated by the bunch should be smaller than that generated by the laser pulses at the bunch tail. For an electron bunch with Heaviside step-function profiles in radial and axial directions, the beam loading density limit, i.e., when the beam-driven wake amplitude equals the amplitude of the laser-driven transverse wakefield, can be written as (see Methods)

4 $${n}_{bl}=\frac{4\,C{a}_{u}}{{k}_{p}^{2}{w}_{0}{r}_{b}\mathrm{(1}-\,\cos \,{k}_{p}{L}_{b}){K}_{1}({k}_{p}{r}_{b}){I}_{1}({k}_{p}{r}_{b})}{n}_{p0},$$

where *I*_1_ and *K*_1_ are the first-order modified Bessel function of the first and second kind, *n*_*p*0_ is the on-axis plasma density, *r*_*b*_ is the transverse radius of the bunch and *L*_*b*_ is the longitudinal length of the bunch. As an example, in a plasma undulator created by a fundamental Gaussian mode and a first-order Hermite-Gaussian mode with *a*_1_ = 0.1, *a*_0_ = $\sqrt{2{a}_{1}}$, *C* = 0.38, *λ*_*L*_ = 1 *μ*m, *w*_0_ = 7 *μ*m, *r*_*b*_ = 0.5 *μ*m, *L*_*b*_ = 1 *μ*m, *n*_*p*0_ = 0.001*n*_*c*_, the limit of the electron density *n*_*bl*_ is 16*n*_*p*0_. Figure 6(a) plots different radiation spectra for different bunch densities. When the bunch density approaches the density limit, the undulator radiation becomes weaker as the undulator is suppressed. To keep a high performance of the plasma undulator, the applicable electron bunch density can be chosen as around 1% of *n*_*bl*_, as shown in Fig. 6(a). Theoretically, by the choice of the undulator parameters and electron bunch shaping, the current can approach kA limit. For instance, with the same parameters stated above but higher laser intensities of *a*_1_ = 1.0 and *a*_0_ = $\sqrt{2}$, the 1% of the beam loading limit *n*_*bl*_ reaches 0.008*n*_*c*_, which corresponds to an electron number of 0.6 × 10^7^ and a current of 0.3 kA.

From the simulation, one can obtain the peak energy radiated per unit solid angle per unit frequency interval *d* ^2^ *I*/*dωd*Ω = 10^−29^ J/*Hz* for one electron. Then the brightness of the radiation emitted by the electron beam in the simulation can be estimated as 10^19^ photons/(s mrad^2^ mm^2^ 0.1%BW), while the electron bunch density here is 0.01 *n*_*p*0_ (see Methods for the calculation of the electron radiation). When the bunch density increases to 0.16*n*_*p*0_ (1% of *n*_*bl*_), the radiation brightness increases to 7 × 10^19^ photons/(s mrad^2^ mm^2^ 0.1% BW). The dependence of the brightness on the laser intensity is presented in Fig. 6(b), where the bunch density is chosen as 1% of *n*_*bl*_. With laser pulses with amplitudes *a*_1_ = 1 and *a*_0_ = $\sqrt{2}$, the peak brightness of the undulator radiation is about 10^22^ photons/(s mrad^2^ mm^2^ 0.1% BW).

## Discussion

It is interesting to compare the main parameters of a permanent magnetic undulator, a plasma undulator and an RF undulator (see Table 1). Plasma undulator has a notably shorter period (about 1 mm or even less by using higher modes) as compared to the other two, whose period is typically more than 10 mm. The undulator strength parameter *K* of the plasma undulator can be tuned in a broad range by changing the laser normalized intensity *a*_0_ of the laser pulse. The main disadvantage of a plasma undulator is the number of periods per segment which is around 20, while the number is on the order of 100 for a magnetic or an RF undulator. Thus a multi-stage scheme (Fig. 4) is very important for the FEL instability to develop. It should be mentioned that when we calculate the shortest wavelength of the radiation from a plasma undulator, we use the highest energy of electrons from an LPA at present^30^.

**Table 1:** Comparison between a magnet undulator, a plasma undulator and an RF undulator.

Parameter	Magnet undulator^*^	Plasma undulator	RF undulator^†^
Number of periods	50 ∼ 200	20 ∼ 30	∼100
Period length	20 ∼ 80 mm	∼1 mm	∼14 mm
Strength parameter	0.7 ∼ 3.0	0.3 ∼ 3.0	0.2 ∼ 0.7
Wavelength range of potential use^‡^	0.05 ∼ 100 nm	0.008 ∼ 100 nm	0.05 ∼ 100 nm
Bandwidth of on-axis radiation	∼1%	∼5%	∼1%

^*^The parameters of a magnet undulator come from FLASH and European XFEL^42^.

^†^The parameters of an RF undulator come from S. Tantawi’s work^43^.

^‡^The radiation wavelength strongly depends on the beam energy.

It is also very interesting to calculate the FEL parameters for the plasma undulator. The ideal 1D power gain length can be calculated by *L*_*g*0_ = *λ*_*u*_/(4*π* $\sqrt{3{\rho }}$), where $\rho =1.78\times {10}^{-5}{A}_{u}^{\mathrm{2/3}}{\lambda }_{u}^{\mathrm{2/3}}[{\rm{cm}}]{n}_{e}^{\mathrm{1/3}}[{{\rm{cm}}}^{-3}]/{\gamma }_{0}$ is the Pierce parameter, *A*_*u*_ = *K*[*J*_0_(*χ*) − *J*_1_(*χ*)]/$\sqrt{2}$ with *χ* = *K* ^2^/(4 + 2*K* ^2^), *K* is the strength parameter and *J*_0_, *J*_1_ are the Bessel functions of the first kind. Using the parameters in the simulation, *λ*_*u*_ = 0.97 mm, *γ*_0_ = 1000, *K* = 0.44, *n*_*e*_ = 0.001 × 10^21^ cm^−3^, we get *ρ* = 1.7 × 10^−3^ and *L*_*g*0_ = 27 mm ≈ 27*λ*_*u*_, which is less than the length of 2 segments of the present plasma undulator. It should be noted that many effects such as electron beam energy spread and emittance, space charge, finite bunch length and radiation diffraction will increase the gain length up to *L*_*g*_ ≈ 2*L*_*g*0_^32^. The power of the radiation *P* ∝ exp(*z*/*L*_*g*_) becomes saturated after a saturation length *L*_*s*_ ≈ 20*L*_*g*_. Therefore, the length of the present plasma undulator required to reach saturation is roughly *L*_*s*_ ≈ 1 m, corresponding to *N*_*s*_ ≈ 1000 undulator periods or 50 plasma undulator segments. We can then estimate the peak brightness of FEL radiation after a saturation length as 10^22^ × exp((*L*_*s*_ − *L*_*u*_)/*L*_*g*_) ≈ 10^30^ photons/(s mrad^2^ mm^2^ 0.1% *BW*), where *L*_*u*_ is the length of one undulator.

The stability of a plasma undulator system is an important issue which should be considered in practical applications. Since a plasma undulator is essentially a wakefield generated by lasers, the stability of a plasma undulator is hence strongly dependent on the laser and plasma stability. The period of the plasma undulator is determined by the laser spot size while the strength parameter *K* is mainly determined by the laser power and plasma density. Note that the fluctuation of *K* caused by the plasma density error is much smaller than that caused by the laser error when the laser duration is around the optimized value, which can be deduced from the expression of *C* parameter in equation (1). Thus the fluctuation of the radiation fundamental wavelength can be expressed as

5 $$\frac{\delta \lambda }{\lambda }=\sqrt{{(2\frac{\delta {w}_{0}}{{w}_{0}})}^{2}+{(\frac{2{K}^{2}}{1+{K}^{2}/2}\frac{\delta P}{P})}^{2}},$$

where *w*_0_ is the radius of the laser spot and *P* is the laser power. For a standalone undulator, its parameters are quite stable because *δw*_0_ ≈ 0 and *δP* ≈ 0 when laser propagating in the underdense plasma. However, for a multiple-stage scheme, the matching between separate undulators becomes important. Technologically, each laser pulse driving an undulator should be generated by beam splitters from a single laser pulse, thus ensuring stability. For the FEL instability to develop, the fundamental wavelength has to be tuned with an accuracy given by *δλ*/*λ* ≤ *ρ*. Thus stable and precise control of beam splitter mirrors and focus mirrors is important for the FEL instability to occur, while shot-to-shot laser fluctuations are less critical as they only change the final radiation wavelength.

In conclusion, we have demonstrated a plasma undulator excited by high-order laser modes in a matched plasma channel, in which high-quality electron beams can make undulator oscillations with a few tens of cycles and emit bright X-ray radiation with a narrow bandwith. The advantages of the plasma undulator rely on matching the intensity relationship between the modes to suppress the betatron oscillation, and tapering the density of the plasma channel to lock the phase of the electron beam in the undulator. The polarization of the radiation can be controlled by changing the phase difference of the modes. The beam loading limit indicates that the tolerated beam charge can lead to currents as high as 0.3 kA, theoretically approaching the kA level. Such a plasma undulator, together with a laser-plasma accelerator, may open the way to realize an extremely compact FEL.

## Methods

### Calculation of the wakefield by two modes

The Hermite-Gaussian laser beams are guided in a matched plasma channel with a parabolic transverse profile as

6 $${n}_{p}(r)={n}_{p0}+{\rm{\Delta }}{n}_{c}\frac{{r}^{2}}{{w}_{0}^{2}},$$

where *r* is the transverse coordinate, *n*_*p*0_ is the on-axis electron density, ${\rm{\Delta }}{n}_{c}={(\pi {r}_{e}{w}_{0}^{2})}^{-1}$ is the critical channel depth, *r*_*e*_ = *e* ^2^/*m*_*e*_ *c* ^2^ is the classical electron radius^18^. In 2D, a guided Hermite-Gaussian mode can be expressed as^31^

7 $${a}_{(m)}(x,z)=\frac{{a}_{m}}{{(m{!2}^{m})}^{1/2}}{H}_{m}(\frac{\sqrt{2}x}{{w}_{0}})\exp [\frac{-{x}^{2}}{{w}_{0}^{2}}+i{\theta }_{m}],$$

where *H*_*m*_ is the Hermite polynomial of order *m* and ${\theta }_{m}=(-z\mathrm{/2}\,{k}_{L})[{k}_{p}^{2}+\mathrm{2(2}\,m+\mathrm{1)/}{w}_{0}^{2}]$ is the phase, *k*_*L*_ is the laser wave number and *k*_*p*_ is the plasma wave number. From the phase *θ*_*m*_ one can see that different modes propagate with different phase velocities. The phase speed for a specific mode *m* is ${v}_{ph}/c\simeq 1+[{k}_{p}^{2}+\mathrm{2(2}m+\mathrm{1)/}{w}_{0}^{2}\mathrm{]/2}{k}_{L}^{2}$. Consider two modes with the same linear polarization (*y* direction) and the same frequency, the total normalized intensity reads

8 $$I={a}_{\perp }{a}_{\perp }^{\ast }={|{a}_{y,{m}_{0}}|}^{2}+|{a}_{y,{m}_{1}}{|}^{2}+2|{a}_{y,{m}_{0}}||{a}_{y,{m}_{1}}|\,\cos [\frac{({m}_{0}-{m}_{1})z}{{Z}_{R}}].$$

One can see that the total intensity oscillates with a wavelength of 2*πZ*_*R*_/|*m*_0_ − *m*_1_|. The wakefield generated by the two modes will also oscillate while propagating in the matched channel. Assuming two modes with mode numbers *m*_0_ (even) and *m*_1_ (odd) and with the same Gaussian profiles in all dimensions (i.e., $I\propto \exp [-{\zeta }^{2}\mathrm{/(2}{L}^{2})]\,\exp \,\,(-{r}^{2}/{w}_{0}^{2})$) and ${a}_{\perp }{a}_{\perp }^{\ast } < 1$, the laser-excited, normalized wake potential is^18^

9 $${\varphi }(\zeta ,x,z)=-C{a}_{\perp }{a}_{\perp }^{\ast }\,\sin \,({k}_{p}\zeta ),$$

and

10 $$\vec{E}/{E}_{0}=-\nabla {\varphi }/{k}_{p},$$

yielding equation (1). Note that the second and higher order terms are omitted here, which implies that the area of interest is close to the axis (${x}^{2}\ll {w}_{0}^{2}$).

### Particle-in-cell simulations

The simulations have been performed on JURECA^39^ at Jülich Supercomputing Centre using 2D/3D PIC code LAPINE^40^. Two *y*-polarized laser modes, the fundamental Gaussian mode and the first-order Hermite-Gaussian mode, propagate into a plasma channel from the left boundary. The normalized peak intensities are *a*_0_ = 0.85 × 10^−9^ $\sqrt{{I}_{L}({W/\mathrm{cm}}^{2}){l}_{L}({\mu }{\rm{m}})}$ = 0.14 for the fundamental Gaussian mode and *a*_1_ = 0.1 for the first-order Hermite-Gaussian mode, respectively. The spot radius *w*_0_ = 7*λ*_*L*_, duration FWHM (full width at half maximum) *τ* = 15 *T*_*L*_, laser wavelength *λ*_*L*_ = 1 *μ*m and laser period *T*_*L*_ = 3.33 fs are the same for the both. The transverse profile of the plasma channel is designed according to equation (6), in order to guide the laser pulses. The plasma density along the propagation axis is *n*_*p*0_ = 0.001*n*_*c*_, where ${n}_{c}={m}_{e}{\omega }_{L}^{2}\mathrm{/(4}\pi {e}^{2})=1.1\times {10}^{21}{\lambda }_{L}(\mu {\rm{m}})/{\rm{c}}{m}^{3}$ is the critical density. Test electrons with *γ*_0_ = 1000 are injected into the plasma channel at a longitudinal position with phase *k*_*p*_ *ζ* = −5/2 *π*. For the electron beam, the transverse density distribution is ${n}_{b}(x)={n}_{b0}\,\exp (-{x}^{2}/2{\sigma }_{x}^{2})$ with *σ*_*x*_ = 0.5 *μ*m and *n*_*b*0_ = 0.01 *n*_*p*0_, and the density distribution in the momentum space is ${n}_{b}({p}_{x}/{m}_{e}c)={n}_{b0}\,\exp [-{({p}_{x}/{m}_{e}c)}^{2}\mathrm{/2}{\sigma }_{px}^{2}]$ with *σ*_*px*_ = 0.05, corresponding to normalized emittance *ε*_*n*_ = 0.025 *μ*m. The rms energy spread of the electron beam is *σ*_*γ*_/*γ*_0_ = 1% with *γ*_0_ = 1000. The density distribution of the electron beam in longitudinal direction is uniform with a length of *L*_*b*_ = 1 *μ*m. In the 2D simulations, a moving window consisting of 600(*x*) × 1800(*z*) grids with 9 electrons and 9 protons per cell is used to follow the long-distance propagation. The resolutions are *dx* = 0.1 *λ*_*L*_ and *dz* = 0.05 *λ*_*L*_. In the 3D simulations, the laser pulse mixture propagates only 2700 laser periods because of the limitation due the computing time. The size of the simulation box is 30*λ*_*L*_(*x*) × 30*λ*_*L*_(*y*) × 90*λ*_*L*_(*z*) corresponding to grids 150(*x*) × 150(*y*) × 900(*z*), with 8 macro-particles per cell for the background plasma and 64 macro-particles per cell for the injected electrons.

In the plasma channel, to maintain a constant phase in the transverse field the plasma density has been tapered as^33^

11 $$\frac{d\sqrt{{n}_{p0}/{n}_{c}}}{d(z/{\lambda }_{L})}=2\pi \frac{{n}_{p0}/{n}_{c}}{|{\psi }_{0}|}[\frac{1}{2{\pi }^{2}{({w}_{0}/{\lambda }_{L})}^{2}}-\frac{{n}_{p0}/{n}_{c}}{4}],$$

where *ψ*_0_ = *k*_*p*_ *ζ*(*z* = 0, *t* = 0) is the initial phase.

### Calculation of the wakefield by an electron beam

According to the theory of beam-driven plasma wakefield^37,38^, the transverse field excited by an electron beam with a number density *n*_*b*_(*r*, *ζ*) = *n*_*b*0_ *ψ*(*r*)*f*(*ζ*) can be expressed as

12 $${W}_{\perp }(r,\zeta )=4\,\pi e{k}_{p}{n}_{b0}{\int }_{0}^{\zeta }d\zeta ^{\prime} {\int }_{0}^{\infty }r^{\prime} dr^{\prime} {\partial }_{r^{\prime} }\psi (r^{\prime} ){I}_{1}({k}_{p}{r}_{ < }){K}_{1}({k}_{p}{r}_{ > })f(\zeta ^{\prime} )\sin \,{k}_{p}(\zeta ^{\prime} -\zeta ),$$

where *I*_1_ and *K*_1_ are the first-order modified Bessel function of the first and second kind, *ψ*(*r*) and *f*(*ζ*) are the density profiles in radial and axial directions, respectively, *r*_<_ = min(*r*, *r* ^'^) and *r*_>_ = max(*r*, *r* ^'^). For a electron bunch with Heaviside step-function profiles in both radial and axial directions, $\psi (r)= {\mathcal H} ({r}_{b}-r),f(\zeta )= {\mathcal H} (-\zeta ) {\mathcal H} (\zeta +{L}_{b})$, *r*_*b*_ is the radius of the electron bunch and *L*_*b*_ is the longitudinal length of the electron bunch, the radial wakefield in the body of the bunch (*r* ≤ *r*_*b*_, −*L*_*b*_ ≤ *ζ* ≤ 0) can be written as

13 $${W}_{\perp }(r,\zeta )={E}_{0}{k}_{p}{r}_{b}\frac{{n}_{b0}}{{n}_{p0}}\mathrm{(1}-\,\cos \,{k}_{p}\zeta ){I}_{1}({k}_{p}r){K}_{1}({k}_{p}{r}_{b}\mathrm{).}$$

Let the amplitude of *W*_⊥_ equal to the amplitude of the transverse wakefield in equation (1), one can obtain the beam loading limit in equation (4).

### Calculation of the electron radiation

The radiation is calculated using the trajectories from PIC simulations. We computed the radiation spectra in the far field by^41^

14 $$\frac{{d}^{2}I}{d\omega \,d{\rm{\Omega }}}=\frac{{e}^{2}}{16\,{\pi }^{3}{\varepsilon }_{0}c}{|{\int }_{-{\rm{\infty }}}^{{\rm{\infty }}}\frac{\overrightarrow{n}\times [(\overrightarrow{n}-\overrightarrow{\beta })\times \dot{\overrightarrow{\beta }}]}{{(1-\overrightarrow{\beta }\overrightarrow{n})}^{2}}{e}^{i\omega (t-\overrightarrow{n}\overrightarrow{r}/c)}dt|}^{2}.$$

The radiation spectra of the electron beam is calculated by incoherent adding all the spectra of the 1100 macro-particles. The estimation of the radiation peak brightness [in unit of photons/(s mrad^2^ mm^2^ 0.1% BW)] is as follows. We first multiply the term *d* ^2^ *N*_*ph*_/*dω d*Ω of one electron by the total electron number ${n}_{b}\pi {r}_{b}^{2}{L}_{b}$ in the bunch, then we divide it by the bunch duration *L*_*b*_/*c* and the sectional area $\pi {r}_{b}^{2}$, and then multiply it by 0.1% *BW* and 10^−6^ due to conversion from square radians to squared milliradians.

### Data availability

The data that support the findings of this study are available from the corresponding authors upon request.

## Electronic supplementary material

Supplementary Information
