# Supplementary material for: Plasma channel undulator excited by high-order laser modes
Source: Sci Rep. 2017 Dec 4;7:16884. doi: 10.1038/s41598-017-16971-5 (PMC5715075; doi:10.1038/s41598-017-16971-5)
Supplement: Supplementary file 1 — Supplementary Information [file 41598_2017_16971_MOESM1_ESM.pdf]

Plasma channel undulator excited by high-order laser modes

J. W. Wang,[1] C. B. Schroeder,[2] R. Li,[3] M. Zepf,[1,4,5] and S. G. Rykovanov[1]

[1]Helmholtz Institute Jena, Fröbelstieg 3, 07743 Jena, Germany

[2]Lawrence Berkeley National Laboratory, 1 Cyclotron Road, Berkeley, California 94720, USA

[3]Shanghai Institute of Optics and Fine Mechanics, Chinese Academy of Sciences, Shanghai, China

[4]Institut für Optik und Quantenelektronik, Friedrich-Schiller-Universität Jena, Max-Wien-Platz 1, 07743 Jena, Germany

[5]Department of Physics and Astronomy, Queen's University Belfast, Belfast BT7 1NN, UK

The spectrum and transverse distribution of the undulator radiation

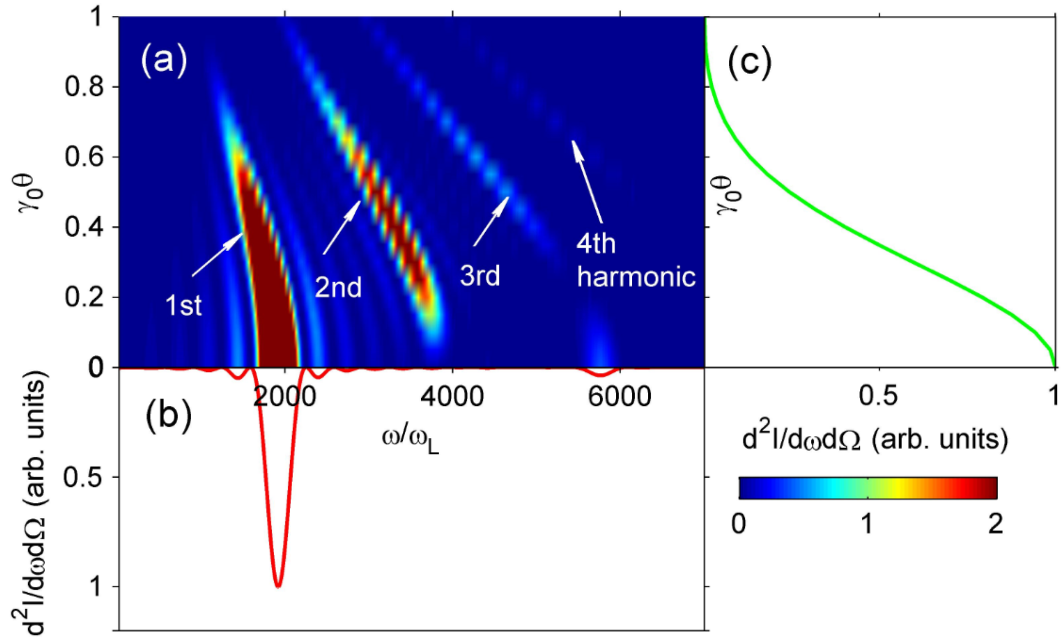

Supplementary Figure 1. The spectrum and transverse distribution of the undulator radiation. (a) The radiation distribution in the space $(\omega/\omega_L, \gamma_0\theta)$. The laser parameters of the laser pulses and plasma are the same as in Fig. 2 in the main manuscript. θ is the azimuthal angle with respect to the propagation direction of electrons. The color here represents the normalized brightness $d^2I/d\omega d\Omega$ (arb. units) of the radiation. (b) The spectrum of the on-axis radiation. On axis only the odd harmonics are observed with the frequencies $\omega_m = m\,2\gamma_0^2\omega_u/(1+K^2/2), m = 1, 3, 5, \ldots$, while both odd and even harmonics are generated off axis with the frequencies $\omega_m(\theta) = m\,2\gamma_0^2\omega_u/(1+K^2/2+\gamma_0^2\theta^2), m = 1, 2, 3, \ldots$ (c) The dependence of the intensity of the first harmonic on the normalized azimuthal angle. The angular width of the first harmonic is $\sigma_\theta \approx 1/(\gamma_0\sqrt{N_u})$, with N_u the period number of the undulator.
